# X-Rays in the Treatment of Superficial Cancers

**Published:** 1903-04

**Authors:** Alfred L. Gray

**Affiliations:** Richmond, Va., Professor of Physiology in the University College of Medicine


### X-RAYS IN THE TREATMENT OF SUPERFICIAL CANCERS.*

BY ALFRED L. GRAY, M.D., RICHMOND, VA.,
Professor of Physiology in the University College of Medicine.

That the Roentgen ray is an agent of the most powerful therapeutic value in at least some forms of skin disease has, by all who have had any considerable experience in this method of treatment, ceased to be a matter of doubt.

Of the graver forms of skin affections, the treatment of which by

* Read before the Richmond Academy of Medicine and Surgery, February 24, 1903.

radiotherapy has proven successful, I mention especially lupus vulgaris and erythematosus, psoriasis, xeroderma pigmentosum, eczema resisting other therapeutic agents and epithelioma.

Since the subject assigned to me is limited, I shall briefly discuss the treatment of the more superficial forms of cancer. The forms in which the most uniformly good results have been reported have been those forms amenable to treatment by excision with the knife, the application of caustic pastes and of the actual cautery. This fact caused the belief that, in order to obtain beneficial results, an actual "burn" must be produced; consequently, in the treatment of cases by the X-ray, the length of exposure necessary for a tube of definite resistance to produce this effect was ascertained and an attempt was made to produce a burn of gradually increasing severity until a certain depth was reached. This was indeed a most tedious and time-consuming method of cauterizing. Further experimentation, however, has demonstrated conclusively the fallacy of this theory. The exact *modus operandi* of the rays on the diseased tissues has furnished a subject for much speculation, some being of the opinion "that the effect is produced by the projection of molecules into the tissues"; some, that electrical discharges or waves emanating from the tube are the active agents; some, that the tissue elements are made to vibrate in a manner different from their normal molecular motion, with a resulting molecular disintegration; some, that the ultra-violet ray is produced "within the tissue by a process of interference"; others, that ozone is liberated in the tissues by the X-rays.

The most generally accepted theory is that the "rays are composed of negatively charged corpuscles or electrons." The theory of the bactericidal action of the rays has been practically abandoned. Whatever be the correct theory, the fact remains, as shown by recent experiments of Dr. Ellis of the Jefferson Hospital, that a distinct and marked degeneration results in the diseased tissues subjected to the action of the rays, resulting in a replacement of the malignant tissue by fibrous or adipose tissue, and this change may be produced without subjecting the patient to the suffering and inconvenience, not to mention the scars, resulting from sloughing of tissue incident to a burn.

Our object being to produce a cure *tuto, cito et jucunde*, if the cure is as certain and the after appearance of the diseased area better, we may sacrifice rapidity for safety, pleasantness and good cosmetic effect. The idea of producing an actual destruction of tissue by the so-called X-ray burn has therefore been abandoned. Authorities differ in the amount of "reaction" desirable, but it seems generally preferable to make the exposures long enough to produce a slight hyperemia, as is seen from exposure to the sunlight, and as in the case of exposure to sunlight, a subsequent "tanning."

It is not my desire to go to any length into the technique of the treatment of these cases, but a few practical points may be at least referred to. The healthy skin surrounding the diseased area must be protected by some substance more or less opaque to the rays. The materials most commonly used are lead foil and rubber sheeting (1-16 to ¼ inch in thickness). An opening is cut into the protective sufficiently large to expose the diseased area and a small band of healthy skin surrounding it. Rubber is to be preferred, as it does not attract electricity, and the patient is therefore less liable to slight shocks that are sometimes experienced when foil is used. Short exposures should be made at first, five to ten minutes or less, at a distance of eight to ten inches from the anti-cathode or platinum plate. These should be made every third day for a series of four or six exposures, and an interval of ten days or two weeks allowed to elapse in order to determine whether or not the individual possesses any unusual susceptibility to the rays. After this the length of exposures should be gradually increased if no ill effects are present, and the distance from the tube reduced to six inches or less.

The effects of treatment become manifest within time limits varying from almost immediately to six or eight weeks, and should be kept up as long as any improvement whatever is noticeable and for some time after healing is apparently complete. The first effect is relief to a greater or less extent of pain. Then follows lessening of the discharge, if such be present, a drying up of the diseased tissue and scaling off in the form of a crust, the lesion contracting at the periphery, and an ultimate *restitutio ad integrum* with scarcely a resulting scar.

As in every other diseased condition there are cases of external cancer that defy this as well as all other forms of treatment. Thrice happy would we be if we possessed a single remedy that would be an absolute specific in every case of any disease. If then, in a malady of such a serious nature as carcinoma, wherever its location, we discover an agent that effects a cure in a large percentage of cases, is it not worthy of a most careful and thorough consideration?

The cases least favorable for treatment by radiotherapy are those that have progressed until the whole system has, to a great extent, lost its vigor, and the vitality of the tissues is so impaired that their reparative power is forever gone. However, this is an almost indeterminable problem, as has been shown by the recovery of many cases considered hopeless. Is there any case, then, that is not worth the trial?

It is well to bear in mind that not infrequently, from too short exposures, the diseased tissues are stimulated and the progress of the malady hastened. The remedy should therefore be faithfully and skilfully administered lest evil rather than good result.

If, gentlemen, in this brief and unsystematized paper I have succeeded in arousing your interest in this most interesting subject, I shall consider myself amply repaid.

REFERENCES.

Williams—The X-ray in Medicine and Surgery. Zeisler—Radiotherapeutic Observations (*Medical Journal*, Philadelphia, February 21, 1903). Franklin—The X-Ray (*Medical Record*, October 25, 1902). Newcomb—*Philadelphia Medical Journal*, January 10, 1903. Leonard—*Ibid.*, February 14, 1903. Hyde and Montgomery—*Journal American Medical Association*, January 3, 1903. Allen—Monograph: The Present Status of Radiotherapy in Cutaneous Disease and Cancer. Hall-Edwards—*Archives of the Roentgen Ray*, December, 1902.

